# Comment on: Clinical and Functional Outcomes of Ilizarov Bone Transport in Traumatic Tibial Bone Loss

**DOI:** 10.5704/MOJ.2511.022

**Published:** 2025-11

**Authors:** G Alibakan, Y Sulek

**Affiliations:** 1 Department of Orthopedics and Traumatology, Sisli Hamidiye Etfal Training and Research Hospital, Istanbul, Turkey; 2 Department of Orthopedics and Traumatology, Esenyurt Necmi Kadioglu State Hospital, Istanbul, Turkey

Dear editor,

We read with interest the recent article by Mohd-Yusof *et al*^1^, which reported excellent union rates (95%) and high proportions of good-to-excellent ASAMI bone (90%) and functional (90%) outcomes in patients with traumatic tibial bone loss treated with Ilizarov bone transport, with 82.5% returning to work. The authors also demonstrated a strong correlation between functional outcomes and return-to-work rates, highlighting the socio-economic impact of successful reconstruction.

In our recent comparative study of cable-assisted bone transport (CASt) versus circular external fixator-assisted bone transport (CEFt) for tibial bone defects, both techniques achieved similar radiological consolidation and ASAMI functional outcomes^2^. However, CASt was associated with significantly lower pain scores during distraction (VAS 4.81±0.98 vs 6.75±0.86 ; p=0.001) and a reduced incidence of pin-tract infections (50% vs 93.8% ; p=0.013) compared to CEFt. These findings suggest that less invasive constructs may enhance patient comfort and potentially reduce complication-related treatment interruptions.

When comparing the two reports, both our series and Mohd-Yusof *et al’s* ^1^ cohort achieved union rates exceeding 90% and similarly high functional scores, reaffirming the efficacy of distraction osteogenesis in large tibial defects. The notable difference lies in complication profiles: while pin-tract infection occurred in 30% of cases in the Ilizarov series, our CEFt group experienced 93.8%, and our CASt group 50%. Although differences in patient selection, defect size, and follow-up protocols limit direct comparison, these observations support continued investigation into transport techniques that minimise transosseous pin usage.

While Mohd-Yusof *et al*^1^ did not stratify outcomes by defect size, previous studies have indicated that larger defects may be inversely correlated with functional recovery and return-to-work potential. This dimension, alongside the choice of transport technique, could influence long-term rehabilitation and socio-economic reintegration, and therefore warrants further exploration in future multicentre studies.

We commend the authors for emphasising return-to-work as a meaningful outcome, a parameter often overlooked in reconstructive orthopaedics. Future prospective comparative trials directly evaluating Ilizarov, CASt, and hybrid methods, while considering defect size as a prognostic factor, may clarify whether the lower morbidity observed with CASt translates into improved long-term function and earlier reintegration into daily and occupational activities.
