# Supplementary material for: Comment on: Clinical and Functional Outcomes of Ilizarov Bone Transport in Traumatic Tibial Bone Loss
Source: Malays Orthop J. 2025 Nov;19(3):152–4. doi: 10.5704/MOJ.2511.022 (PMC12798137; doi:10.5704/MOJ.2511.022)
Supplement: Related texts

# Authors’ Reply to the Letter to the Editor

**Published:** 2025-11

**Authors:** RY Kow, N Mohd-Yusof

**Affiliations:** Traumatology and Rehabilitation, International Islamic University Malaysia, Kuantan, Malaysia

Dear editor,

We thank Alibakan and Sulek for their insightful commentary comparing our series of Ilizarov bone transport with their recent work on cable-assisted bone transport (CASt)^1^. Their contribution highlights the ongoing evolution of distraction osteogenesis techniques aimed at improving patient comfort and reducing complications.

In our study, the Ilizarov method demonstrated union rates of 95% with good-to-excellent ASAMI bone and functional outcomes in 90% of cases, and importantly, 82.5% of patients successfully returned to work^2^. We agree with the authors that while union and function are essential, the socio-economic dimension of return-to-work is an equally critical endpoint. The differences in complication profiles observed between CASt, CEFt, and Ilizarov techniques underscore the importance of tailoring constructs to optimise both biological success and patient tolerability.

We also acknowledge their point regarding defect size and long-term recovery. Stratification by defect length is indeed an important prognostic factor, and future multicentre comparative studies, including Ilizarov, CASt, and hybrid methods, will be instrumental in clarifying whether the lower morbidity associated with CASt translates into earlier socio-economic reintegration.

We read with interest their observation that CASt achieved similar consolidation and ASAMI functional outcomes compared with circular external fixators, while notably reporting lower pain scores and reduced rates of pin-tract infection (50% vs 93.8%)^1^. It is an interesting point that underscores the potential of this technique to improve patient comfort without compromising biological success. In our series, the pin-site infection rate was 30%, which, while not negligible, remains within the commonly reported range for Ilizarov applications^2^. It is worth noting that infection rates in the literature vary widely^3-5^. Even within the same country, China has reported rates ranging from 21.3% in a large 282-patient cohort to 61.3% in a 199-patient study^3,4^. Rates were even higher at 71.4% in a smaller Indian series5. Such variation likely reflects differences in study size, patient demographics, socioeconomic status, hygiene protocols, and definitions of minor versus major infection.

In our experience, the majority of infections were superficial and responded well to local care and oral antibiotics, without compromising union or functional outcomes. This observation is consistent with larger reports, where minor infections are often self-limiting, while major infections requiring unplanned surgical revision are relatively rare^3,4^. Risk factors such as larger bone defects, multiple previous surgeries, and higher external fixation index (EFI) have been shown to predispose to infection^4^. Conversely, younger patients with smaller tibial defects appear to have lower risk, likely due to stronger immune response and better soft tissue compliance^3^.

Despite these complications, the Ilizarov method in our cohort achieved a 95% union rate, 90% good-to-excellent ASAMI outcomes, and 82.5% return-to-work^2^. These results suggest that, while pin-site morbidity is a limitation, it rarely jeopardises the ultimate goals of union and functional restoration. At the same time, we concur that reducing hardware-related complications, either through techniques such as CASt or hybrid constructs, represents an important future direction.

We commend the authors for advancing this discussion. We believe that ongoing comparative work across centres and techniques strengthens the field by offering surgeons more refined strategies to balance union, function, complication avoidance, and patient quality of life.

## References

[1] Alibakan G, Kanar M, Armagan R, Sulek Y, Altuntas Y, Eren OT (2025). Cable-asisted bone transport versus circular external fixators-asisted bone transport in the management of bone defects of the Tibia: clinical and imaging results.. J Orthop Surg Res.

[2] Mohd-Yusof N, Kow RY, Sulong AF, Nallayan RM, Zainal-Abidin MA. (2025). Clinical and Functional Outcomes of Ilizarov Bone Transport in Traumatic Tibial Bone Loss.. Malays Orthop J.

[3] Liu Y, Yushan M, Liu Z, Liu J, Ma C, Yusufu A (2020). Complications of bone transport technique using the Ilizarov method in the lower extremity: a retrospective analysis of 282 consecutive cases over 10 years.. BMC Musculoskelet Disord.

[4] Feng D, Zhang Y, Jia H, Xu G, Wu W, Yang F (2023). Complications analysis of Ilizarov bone transport technique in the treatment of tibial bone defects-a retrospective study of 199 cases.. BMC Musculoskelet Disord.

[5] Meleppuram JJ, Ibrahim S (2016). Experience in fixation of infected non-union tibia by Ilizarov technique - a retrospective study of 42 cases.. Rev Bras Ortop.
